# Retrospective clinicopathological study of malignant bone tumors in children and adolescents in Romania – single center experience

**Published:** 2016

**Authors:** RC Petca, S Gavriliu, G Burnei

**Affiliations:** * ”Carol-Davila” University of Medicine and Pharmacy, Bucharest, Romania,; ** Department of Pediatric and Orthopedic Surgery, “Maria Sklodowska Curie” Emergency Hospital for Children, Bucharest, Romania

**Keywords:** malignant bone tumors, osteosarcoma, Ewing sarcoma, children

## Abstract

**Rationale:** There is few data on epidemiology or clinico-pathology of malignant bone tumors in children and adolescents in Romania. These tumors are very rare compared to other malignancies, yet they account for a major source of mortality and morbidity among patients with cancer. Bone tumors often have a similar presentation and clinical approach, but they present individual characteristics that are important for treatment and prognosis.

**Objective:** To describe the characteristics of primary malignant bone tumors in children and adolescents in Romania.

**Methods and Results:** A retrospective analysis of all malignant bone tumors registered at a large referral center, “Maria Sklodowska Curie” Emergency Hospital for Children, between 2005 and 2013 was presented. A total of 146 biopsies and surgical resection specimens were reviewed during this period, and were classified as malignant bone tumors.

There were 91 boys and 55 girls in the series, with a male-female ratio of 1.65:1. The average patient age was 13.32 years (2 to 19). The most common anatomical distribution of the tumors was femur - 32.19%, tibia - 25.34% and humerus - 11.64%. Histologically, we found osteosarcoma in 54.1% of all bone tumors, followed by Ewing’s sarcoma – 30.82% and chondrosarcoma – 8.9%.

**Discussion:** Geographic location did not appear to represent a risk factor for any particular type of bone tumor. Our results were parallel to the findings previously reported in the general literature; the distribution and the epidemiology were similar to those in the other developed and underdeveloped countries. Malignant bone tumors in our country have a high mortality rate, because of the late diagnosis.

## Introduction

Cancer is the second leading cause of death worldwide, after heart disease, and accounts for 23% of all deaths. Although most cancers causing death are carcinomas of lung, prostate and breast, primary malignancy of the bone is ranked as the third leading cause of death in patients with cancer who are younger than 20 years [**1**]. Most pediatric bone tumors are benign; however, malignant bone tumors account for a significant amount of morbidity and mortality among children and adolescents. The diagnosis of malignant bone tumors is hampered by delays in presentation.

Malignant bone tumors represent 3-5% of the cancers diagnosed in children aged 0-14 years and 7%-8% of the cancers in adolescents aged 15-19 years (data from resource-rich medical systems) [**2**,**3**]. Malignant bone tumors are rarely diagnosed in children before the age of five. Incidence increases with age and peaks in late childhood or adolescence around the time of puberty, which suggests a relationship between rapid bone growth and the development of malignancy. 

Malignant bone tumors are histologically heterogeneous, with more than 20 different sub-types, but the majority of those diagnosed in children and adolescents are osteosarcomas (52%) and Ewing sarcomas (34%). In children, males and females have similar incidence of osteosarcoma, but the incidence of Ewing sarcoma is higher in males than in females [**3**]. 

There are about 37 children and adolescents diagnosed with malignant bone tumors, annually in Romania; 60% are osteosarcomas, 24% Ewing sarcomas and 16% other types of rare sarcomas [**4**]. There is limited information regarding the epidemiology of bone tumors in Romania.

There is a significant variability of the age, sex, ethnicity of the patients as well as of tumor location. The typical presenting symptoms are similar for most malignant bone tumors with a few distinct variations between subtypes. The individual biological characteristics of each tumor type are important for treatment and prognosis. 

The management of a child or adolescent with bone sarcoma is best carried out by a multidisciplinary team, in a specialized center. Surgical management is highly specialized and the decision regarding the best approach will depend very much upon the nature of the tumor, its extension, the response to chemotherapy, and the expertise available [**5**]. The survival of patients with malignant bone sarcomas has improved dramatically over the past 30 years, largely as a result of advances in chemotherapy. Before the era of effective chemotherapy, 80% to 90% of the patients with malignant bone tumors developed a metastatic disease despite achieving local control from surgery and died of their disease. It was assumed that the majority of these patients had a subclinical metastatic disease that was present at the time of diagnosis, even in the absence of clinical metastases [**6**].

## Materials and methods

A retrospective review was conducted in a large referral center, Department of Pediatric and Orthopedic Surgery, from “Maria Sklodowska Curie” Emergency Hospital for Children, Bucharest, Romania. Newly diagnosed patients with malignant bone tumors were included in the study, during a period of 9 years (2005–2013). The clinical and pathological data were collected from medical records and reviewed while taking into account the ethical standards. Records were analyzed for patient demographics, age, sex, symptoms, duration of symptoms, site of lesion, histological type of tumor, dimensions of the tumor, type of biopsy, treatment, and outcomes.

**Graph 1:**
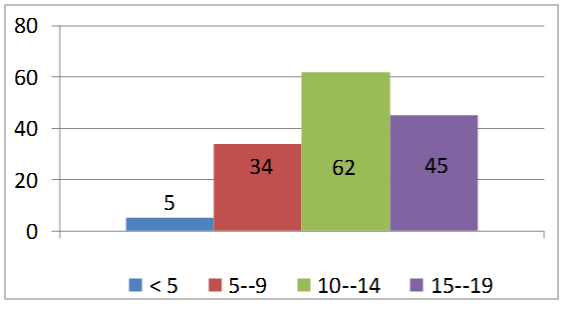
Incidence of malignant bone tumors by age

The study group was composed of 146 of patients with primary bone tumors (biopsies and surgical resection specimens), with ages between 2 and 19 years, median age 13.32, who came from all over the country (**Graph 1**). The majority of patients were from the rural environment – 95/ 146 (65.06%). 

The group was analyzed based on clinical records and laboratory data. The physical examination of all patients included the evaluation of the patient’s general health, as well as a careful examination of the lesion; the mass was measured together with its location, consistency, shape, sensitivity, mobility, tenderness, local temperature, change in position, and joint contractures and skin condition were also noted.

**Fig. 1:**
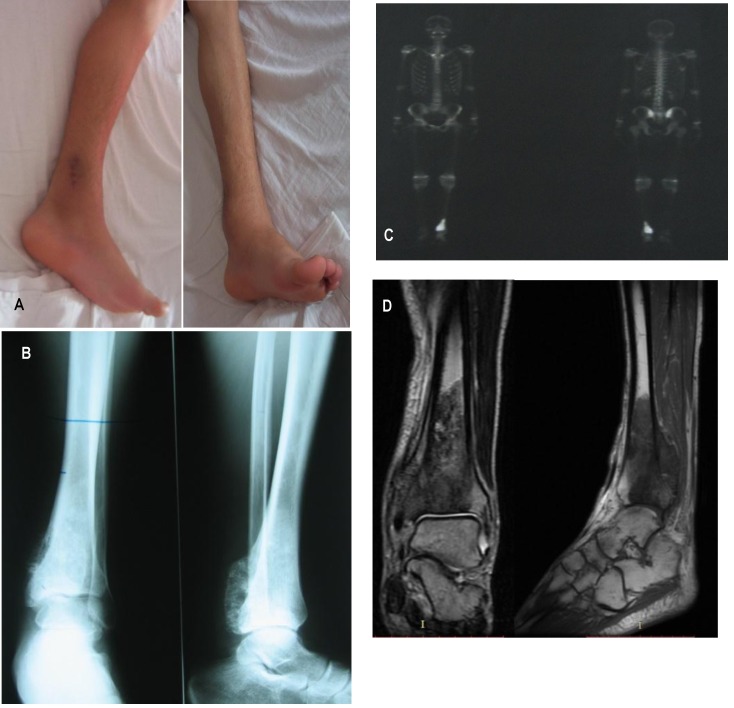
A 17-year-old patient, diagnosed with Ewing sarcoma in the distal third of the left tibia, which invaded the growth cartilage. A. Clinical aspects. B. Preoperative radiological examination. C. Whole body scintigraphy scan - replacement process space well vascularized to the left tibia D. MRI – fusiform heterogeneous tumor mass confined to tibia

All suspected bone neoplasms were initially evaluated with plain radiographs (**Fig. 1**), which was usually a good way to begin the assessment of bone lesions. Plain radiographs can often predict the probable histology of a potentially malignant bone lesion and define the size of the tumor.

All the patients with a suspected primary bone malignancy underwent MRI exams of the entire involved bone (**Fig. 1**), which was the most accurate technique for the determination of the extent of the disease - local intraosseous and extraosseous extent. MRI is the study of choice to determine the size of the tumor, as well as its anatomic relationships to the surrounding tissues [**7**]. CT is the examination of choice for the evaluation of the thorax, which is important because the most common site for metastases from bone sarcomas is pulmonary. Radionuclide bone scanning with technetium is helpful both for diagnosis, staging and for investigating suspicious areas during follow-up. All the examinations were repeated before the definitive surgery. After surgery, chest and primary lesion X-rays, chest CT, and whole-body bone scanning were performed at every 3 months throughout the period of adjuvant chemotherapy. After the completion of the entire treatment course, all the patients were followed at every 3 months during the first 2 years, at every 6 months for the following 3 years and yearly thereafter.

## Results

**Graph 2:**
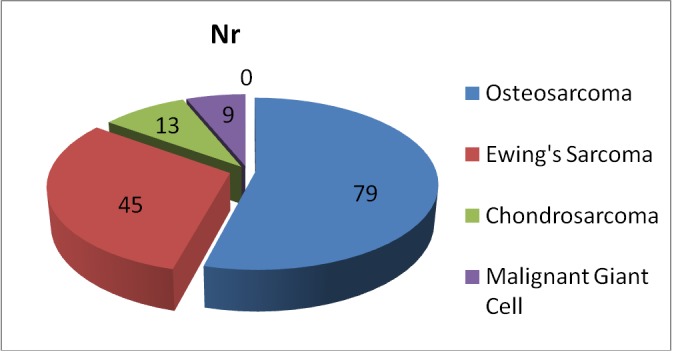
Histologic type of malignant bone tumors

A male to female ratio of 1.65:1 – 91 boys and 55 girls was found. **Graph 2** summarizes the data on the histologic types of malignant bone tumors in children and adolescents in our study. The most common histologic diagnoses were osteosarcoma in 79/ 146 patients (54.1%), followed by Ewing’s sarcoma, in 45/ 146 patients (30.82%), chondrosarcoma in 13/ 146 patients (8.90%) and malignant giant cell tumor – 9/ 146 patients (6.16%). The histologic diagnosis of osteosarcoma depends on the presence of a malignant sarcomatous stroma associated with the production of tumor osteoid or tumor bone. The largest group of osteosarcomas is conventional (intramedullary high-grade) osteosarcomas, which can be classified as osteoblastic, chondroblastic, or fibroblastic, depending on the predominant cellular component. In our series, the distribution of the histologic subtypes of osteosarcoma was osteoblastic – 63.29% (50/ 79 cases), chondroblastic – 10.12% (8/ 79 cases), fibroblastic – 8.86% (7/ 79 cases), telangiectatic - 10.12% (8/ 79 cases) and periosteal – 7.59% (6/ 79 cases).

Duration of symptoms before presentation ranged from 1 month to 3 years. The time until diagnosis was long; in 17.12% (25/ 146) it was less than 3 months, in 34.93% (51/ 146) it was between 3–6 months and in 47.94% (70/ 146) it was more than 6 months. The long period of time between the first symptoms and the moment of diagnosis was found to be a poor prognosis factor. 

The most common presenting symptom was local pain in 92.46%, frequently associated with trauma, often attributable to sports injury. Deep-seated pelvic tumors tended to have a prolonged history, unlike proximal tibia tumors, which tended to have a short history. Other common presenting symptoms included an enlarging soft tissue mass, total or partial functional impotence and pathologic fracture (in 21/ 146 patients - 14.38%). Occasionally, systemic signs such as weight loss, fatigue, anorexia, fever, dyspnea, or night sweating were also found.

The localization of the malignant bone tumors was found to be mainly at the limbs (**Table 1**). Lower limbs were the most frequent site of the lesions. Only a small number of the tumors developed in the pelvic or scapular ring or the vertebral region. We noted the predilection for the distal femur and the proximal tibia or fibula, corresponding to the most active growth plates during the adolescent growth spurt.

**Table 1:** Anatomical distribution of the tumors

Site	Frequency (%)	Site	Frequency (%)
Femur	39 (26.71)	Forearm	8 (5.47)
Tibia	28 (19.17)	Scapular ring	7 (4.79)
Humerus	17 (11.64)	Ribs	6 (4.1)
Foot & ankle	12 (8.21)	Hand & wrist	6 (4.1)
Pelvic ring	9 (6.16)	Vertebra	5 (3.42)
Fibula	9 (6.16)		

The malignant bone tumors were diagnosed based on tumor biopsy reports. Open biopsies were performed in all cases, in order to avoid sampling error and to provide adequate tissue for biologic studies. Most commonly an incisional biopsy was performed – 93.15% (136/ 146 cases) and an excisional biopsy was rarely performed – 6.84% (10/ 146 cases). The incision was carefully planned in order to avoid the tumor contamination of the neurovascular structures and to facilitate the removal of the biopsy tract en bloc during the definitive surgery. No major complications were noted after the biopsy.

The latest AJCC classification of bone sarcomas (Tumor-Node-Metastasis [TNM] system) was based on tumor grade and size and the presence and location of metastases. Stage I tumors were low grade. Stage II tumors were high grade. Stages I and II were subdivided based on the size of the lesion. Stages I-A and II-A were less than or equal to 8 cm in their greatest linear measurement. Stages I-B and II-B were greater than 8 cm in size. Stage III tumors were those that had “skip” metastases, and which were defined as discontinuous lesions within the same bone. Stage IV-A included patients with pulmonary metastases only, whereas Stage IV-B included patients with non-pulmonary metastases (e.g., bone, liver, lymph node) [**8**]. 

An advanced stage of the disease was often found: 17.51% (24/ 137) of the cases were Stage III and 35.76% (49/ 137) cases were stage IV (**Table 2**). An advanced stage of the disease at presentation was found to be a poor prognostic factor. For both osteosarcoma and Ewing sarcoma, most cases were diagnosed in stage II A or IV.

**Table 2:** Staging of tumors at the moment of diagnosis – according to AJCC system for bone sarcomas

Stage	Osteosarcoma	Ewing Sarcoma	Chondrosarcoma	Total
II A	25 (18.24%)	18 (13.13%)	1 (0.72%)	44 (32.11%)
II B	12 (8.75%)	6 (4.37%)	2 (1.45%)	20 (14.59%)
III	14 (10.21%)	6 (4.37%)	4 (2.91%)	24 (17.51%)
IV	28 (20.43%)	15 (10.94%)	6 (4.37%)	49 (35.76%)

Unfortunately, 55 patients were lost from evidence after biopsy or resection. The remaining 91 patients were treated with neoadjuvant and adjuvant chemotherapy, according to the European protocols - COSS 96 for osteosarcoma and EWING 99 for EWING sarcoma. The same protocol was used for preadolescent and adolescent patients. The treatment of chondrosarcoma was primarily surgical; radiotherapy or chemotherapy had not shown any benefit in these cases, except for palliative purposes [**9**]. 

The type of surgery depended on the location and extension of the tumor, neurovascular involvement, and the presence of complications such as pathologic fractures. The reconstruction procedures after tumor resection included the use of prosthesis, autograft, or allograft. Amputation was performed in 35 patients and resection with endoprothesis or reconstruction with autograft/ allograft was performed in 56 patients. The follow-up period varied from 24 to 118 months, the mean follow-up was of 44.5 months. 

The survival rate at 3 years was 70.32% (64/ 91 patients). We did not found any correlation between the therapeutical approach used and the survival prognosis for the patients included in the study.

**Discussion**

Bone tumors are difficult to treat and the mortality rate is high for most histological types. This was the largest series of children and adolescents with malignant bone tumors in Romania, reported in literature. The number of patients was small due to the rarity of these malignancies. The etiology of these often-aggressive neoplasms is currently unknown, and complicated structural and numeric genomic rearrangements in cancer cells preclude the understanding of tumor biology and development.

Osteosarcoma is characterized by an extensive genetic complexity, which is reflected in the similarly complex epigenetic alterations in tumors [**10**]. The mechanisms of genomic instability may be facilitated by the repetitive DNA sequences found in the human genome, particularly low copy repeats. Osteosarcoma is characterized by a high level of genomic instability, in particular one subcategory of instability known as chromosomal instability (CIN) [**11**]. CIN is the elevated rate of gain or loss of entire chromosomes or big parts of chromosomes, and it appears to have a significant role in the pathogenesis of osteosarcoma tumors, resulting in variable structural and numerical aberrations. Genetic studies detected copy number gains on chromosomes 1p, 1q, 6p, 8q, and 17p as well as copy number losses on chromosomes 3q, 6q, 9, 10, 13, 17p, and 18q, but definitive oncogenes or tumor suppressor genes remain elusive with respect to many loci [**12**]. 

The genetic alterations in osteosarcoma are most complex at the molecular level. The pathogenesis is based on the inactivation of tumor suppressor genes, particularly p53 and the retinoblastoma susceptibility gene (RB1). Genetic predisposition plays a role in osteosarcoma. The combination of constitutional mutation of the RB gene (germline retinoblastoma) and radiation therapy is linked with a particularly high risk of developing osteosarcoma, Li-Fraumeni syndrome (germline p53 mutation), and Rothmund-Thomson syndrome (autosomal recessive association of congenital bone defects, hair and skin dysplasias, hypogonadism, and cataract).

The origin of the small round blue cells of Ewing sarcoma remains unknown. Translocation of EWSR1 (Ewing sarcoma breakpoint region 1) with an ETS (E26 transformation-specific) transcription factor gene occurs in more than 95% of Ewing sarcomas. The most common translocation, seen in about 85% of all Ewing tumors, was the t(11;22) (q24;q12) translocation. This translocation joins the Ewing sarcoma gene EWS on chromosome 22 to a gene of the ETS family, friend leukemia insertion (FLI1), on chromosome 11 (ie, t[11;22]). Alternative translocations include EWS-ERG t(21;22), EWS-ETV t(7;22), and EWS-FEV t(2;22), all of which involve the ETS family protein. Recently, approximately 4% of the Ewing sarcoma were identified as having an intrachromosomal X-fusion leading to BCOR (encoding the BCL6co-repressor) and CCNB3 (encoding the testis-specific cyclin B3) [**13**]. No data regarding the cause of the chromosomal translocation is available. Downstream targets and protein partners responsible for EWS-FLI1 transformation of cells are numerous; however, none one of these downstream pathways is either adequate to create a Ewing sarcoma or its inhibition is adequate to lead to Ewing sarcoma cell death [**14**]. Different gene mutations were identified in patients with Ewing sarcoma: TP53 mutations occurred in 5% - 20% of cases, amplifications of MDM2 occurred in up to 10% of the cases, deletions of the CDKN2A in about 15% of the cases [**15**].

As mentioned, literature data on malignant bone tumors in children and adolescents in Romania is little, so we do not know if there was an increase in the incidence over the last period. In our study, we observed that the majority of patients were from the rural environment. Parental farming has been consistently associated with an increased risk of bone tumors in offspring. Valery et al. [**16**] reported the association between farming during pregnancy and malignant bone tumors with an over twofold increased risk, although neither of their odds ratios reached statistical significance. The effect of farming reported by Valery et al. [**16**] was especially pronounced in those diagnosed at ages 0–20 years. To address the inconsistencies of smaller studies, Valery et al. performed a pooled analysis of three case–control studies and found statistically significant associations between both paternal and maternal employment on a farm during the periconceptional and gestation periods.

Osteosarcoma had a high prevalence in malignant bone tumors – 54.1% of the cases included in our study, similar to other studies. High-grade osteosarcoma is the most common bone malignancy in children. Its incidence in the pediatric population varies significantly with age, the peak incidence occurring in the second decade of life, during the adolescent growth spurt [**8**].

In our patients, the most common sites of malignant bone tumors were the long bones of the extremities, with a predilection for the inferior limbs. These findings are in accordance with the previous reports showing that only a small number of cases involve flat bones such as pelvis, rib and skull [**17**,**18**]. No correlation between the site of lesion and patients prognosis was found in our study.

There was a long period of time before establishing the diagnosis (82.87% of the patients with a duration of symptoms longer than 3 months) which, additionally to the advanced stage of the disease, led to an unfavorable prognosis. It is of paramount importance to minimize the time interval between the appearance of symptoms and the start of treatment, because the consequences of a diagnostic delay of 3 months are terrible. It may not be possible to change the behavior and other characteristics of a tumor, but we can improve the time intervals of the diagnostic procedures and avoid delays. The use of screening protocols in oncological centers is mandatory. In addition, we suggest the implementation of educational measures so that general physicians and general orthopedic surgeons would become sufficiently aware of this pathology to send possible patients to reference centers immediately. This will improve prognosis and the functional outcome in pediatric malignant bone tumors.

Advances in the treatment of bone cancers are mainly due to the ongoing collaboration in diagnosis and during each therapeutic stage by oncologists, radiologists, pathologists, and orthopedic surgeons with oncologic specialization. There are improved results due to chemotherapy, which ensure a better overall control and, often, the local disease has allowed surgeons to develop a generally conservative surgery, with the preservation of the limb function and an almost complete elimination of radiotherapy [**19**].

Most chemotherapy regimens applied for osteosarcoma have been based on 4 drugs: high-dose methotrexate with leucovorin rescue, doxorubicin (adriamycin), cisplatin and ifosfamide [**20**]. These agents were integrated into various chemotherapy protocols. Current standard chemotherapy for Ewing sarcoma includes vincristine, doxorubicin, and cyclophosphamide alternating with ifosfamide and etoposide (VDC/ IE) [**21**].

## Conclusion

Our results parallel the findings previously reported in literature and show a similar distribution and epidemiology of pediatric bone tumors as in the other developed and underdeveloped countries. Geographic location does not appear to represent a risk factor for any particular type of bone tumor and does not affect the age distribution, location, or histopathologic type of the lesions. Regardless of age, a delay of 3 months between the appearance of symptoms and the start of treatment causes an increased morbidity and mortality. Hence, efforts need to be focused towards a better information and education of the public, to ensure an early presentation, thereby reducing morbidity and mortality. This is why measures to guarantee an early diagnosis of all the patients who might be suffering from malignant bone tumors are of utmost importance.

**Acknowledgments**

This paper is partly supported by the Sectorial Operational Programme Human Resources Development (SOPHRD), financed by the European Social Fund and the Romanian Government under the contract number POSDRU 141531.
